# The effect of uneven surfaces on inter-joint coordination during walking in children with cerebral palsy

**DOI:** 10.1038/s41598-023-49196-w

**Published:** 2023-12-08

**Authors:** C. Dussault-Picard, Y. Cherni, A. Ferron, M. T. Robert, P. C. Dixon

**Affiliations:** 1 Faculty of Medicine, School of Kinesiology and Physical Activity Sciences, University of Montreal, Montreal, Canada; 2 Research Center of the Sainte-Justine University Hospital (CRCHUSJ), Montreal, Canada; 3 Department of Biology, University of Quebec in Montreal, Montreal, Canada; 4 Department of Rehabilitation, Faculty of Medicine, Laval University, Quebec City, Canada; 5 Institute of Biomedical Engineering, Faculty of Medicine, University of Montreal, Montreal, Canada

**Keywords:** Paediatric research, Movement disorders

## Abstract

Clinical gait analysis on uneven surfaces contributes to the ecological assessment of gait deviations of children with spastic cerebral palsy (CP). Walking on uneven surfaces requires specific motor strategies, which can be assessed by lower-limb kinematic and inter-joint coordination analyses. This study aimed to assess and compare kinematics and inter-joint coordination between children with CP and their typically developing (TD) peers when walking on even and two levels of uneven surfaces (medium and high). A total of 17 children with CP and 17 TD children (11.5 ± 3.5 and 10.4 ± 4.5 years old, respectively) were asked to complete 6–8 gait trials on a 4-m walkway of each surface (n = 3) in randomized blocks while fit with retro-reflective markers on their lower-limbs. Children with CP showed proximal gait adaptations (i.e., hip and knee) on uneven surfaces. Compared with the TD group, the CP group showed decreased hip extension during late stance (49–63%, d = 0.549, *p* < 0.001), and a more in-phase knee-hip coordination strategy during swing phase (75–84% of gait cycle, d = 1.035, *p* = 0.029 and 92–100%, d = 1.091, *p* = 0.030) when walking on an uneven (high), compared to even surface. This study provides a better understanding of kinematic strategies employed by children with spastic CP when facing typical daily life gait challenges. Further studies are needed to evaluate the benefits of integrating uneven surfaces in rehabilitation care.

## Introduction

Cerebral palsy (CP) is characterized by posture and movement disorders caused by a lesion on the developing brain^1^, that contributes to gait deviations. These gait deviations are associated with functional challenges (e.g., reduced balance and fear of falling) that contribute to a decreased participation level in various environments and contexts, compared to typically developing (TD) children^2^. Indeed, the ability to negotiate uneven surfaces is essential for maximizing a child's engagement in their environment.

Clinical gait analysis contributes to assessment of gait deviations, allows for comparison with non-pathological populations^3,4^, and supports orthopedic and pharmacologic decision-making^5^; however, analyses are habitually performed on an even laboratory walkway, which may not allow for the assessment of real-world locomotion challenges. Indeed, this gait assessment may overlook problems only present during challenging walking conditions, such as on uneven surfaces^6^. Gait analysis on uneven surfaces can reveal insights into the functional limitations specific to children with CP when they interact with more challenging environments (e.g., when walking in parks and recreational areas), leading to a better understanding of their daily challenges. Moreover, identifying functional limitations during walking on uneven surfaces would enable the development of more personalized interventions tailored to the child's specific and real-world needs.

A recent scoping review has reported that individuals with CP present different adaptations in comparison to healthy controls when walking on uneven surfaces, such as a greater increase in knee and hip flexion during swing phase^7^. The limited motor control of individuals with CP was highlighted as the potential cause of their impaired ability to simultaneously adapt motion across multiple joints^8,9^. To date, no study has delved into the motor control strategies used by children with CP to adapt gait to different levels of uneven surface. Moreover, the effect of the level of unevenness has never been explored.

Human walking requires multi-joint coordination, analysis of a single joint kinematics may not be sufficient to reveal gait motor control impairments. Gait motor control may be investigated with lower limb coordination, which represents the temporal and sequential organization of the numerous degrees of freedom (e.g., motor units, muscles, segments, joints) into a smooth and controlled motor activity (i.e., a low-degree-of-freedom mechanical model)^10,11^. It has been reported that children with CP exhibit more coordinated inter-joint motion than their TD peers, on even surfaces, which is associated to a more restricted and rigid walking pattern^12–14^. Thus, lower-limb coordination enables the evaluation of gait motor control impairments that account for the observed gait deviations^15^ and provides a distinct approach to interpret the underlying precursor gait kinematic curves.

Thus, this study aimed to (1) assess sagittal kinematics and inter-joint coordination of the lower limbs when walking on uneven surfaces in children with CP and quantify the effect of the different levels of unevenness, and to (2) compare differences with their TD peers. It was hypothesized that children with CP will increase their hip and knee flexion and have a more in-phase coordination pattern (i.e., rigid motor pattern) when walking on uneven surfaces. Furthermore, it is expected that children with CP will exhibit more in-phase inter-joint coordination than their TD peers when walking on uneven surfaces, and that their adaptive responses/mal-adaptations will be amplified by increasing levels of unevenness.

## Methods

### Subjects

Participants aged between 4 and 17 years old were recruited at the rehabilitation center of the Sainte-Justine University Hospital. Inclusion criteria for the participants with CP were a confirmed diagnosis of predominant spastic CP, regardless of type (i.e., unilateral, bilateral), a GMFCS level of I or II, the capacity to understand and follow verbal instructions, and the ability to walk on uneven surfaces without assistive device except orthosis. For TD participants, exclusion criteria were any history of gait, neurological, or musculoskeletal abnormalities. All methods were approved by the Research Ethics Board of Sainte-Justine Hospital (2022–3349) and were carried out in accordance with their guidelines. The written informed consent was given by the parents/guardians.

### Data collection

#### Procedure

**Figure 1:**
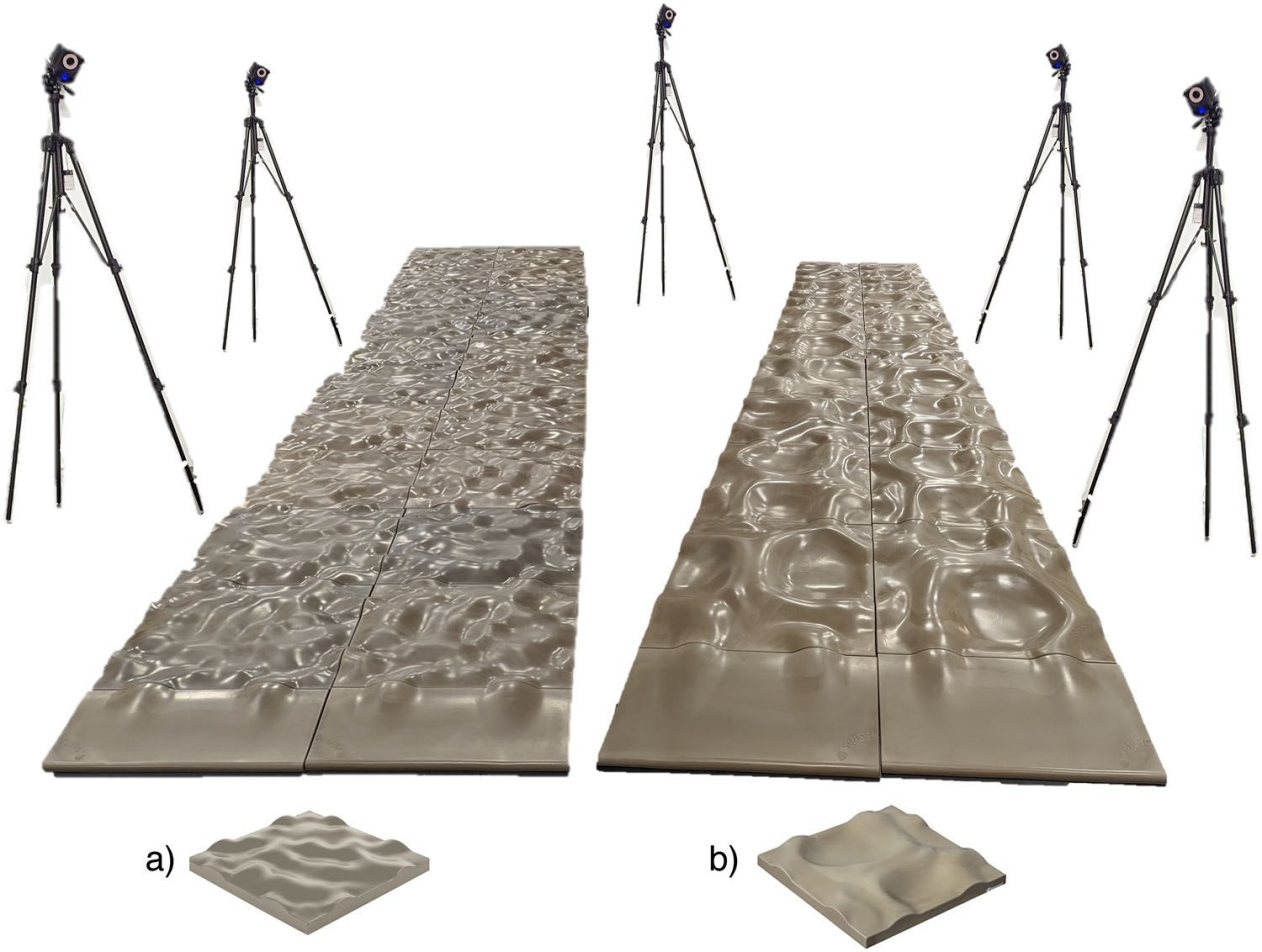
Example of the laboratory setting with the two different levels of uneven surface (**a**) medium, (**b**) high. To facilitate smooth entry and exit onto the uneven surface, two ramps purposefully designed were positioned on either side of the lane.

The participants’ spasticity and joint contractures were first assessed by a highly trained physiotherapist using a Modified Ashworth Spasticity test^16^ and goniometric measurements of the passive range of motion^17^, respectively. Then, participants were asked to walk with their usual shoes and orthosis, if worn daily, along a 4-m walkway at their comfortable gait speed on the lab surface (even), and 2 levels of uneven surface (medium, high) (see Fig. 1). Gait trials were initiated and terminated 3 m before and after the uneven surface to prevent acceleration and deceleration in the data collection area. A total of 6–8 gait trials (i.e., 3 to 4 back-and-forth) per surface were conducted in randomized blocks.

#### Surfaces specifications

The uneven walkways comprised 2 × 8 patented polyurethane floor panels (Terrasensa, Kassel, Germany)^18^. The floor panel was shock-absorbing (i.e., shore hardness of 45A), and has a maximum vertical variation (vertical distance between the lowest and highest points of the surface) of 2 cm for the medium and 5 cm for the high uneven surface.

#### Gait analysis specifications

Reflective markers were positioned on the participant according to the pyCGM (version 2.3) model. This model integrates recent methodological advancements into the standard clinical gait model, including accounting for segmental soft tissue artifacts as well as enhancing the accuracy and reliability of joint kinematic analysis^19^. Trajectories were recorded at 100 Hz via a 12-camera motion capture system (Vicon Motion Systems Ltd., Oxford, UK).

#### Initial data processing

Initial data processing (i.e., marker labelling, gap filling, joint kinematics calculations) was conducted using Vicon Nexus (v2.12.0, Vicon Motion Systems Ltd., Oxford, UK). The trials were exported and further processed in MATLAB (vR2022b, Mathworks Inc., Natick, USA) using the open-source biomechZoo toolbox (v.1.9.10)^20^ and custom code. Gait events (i.e., foot strike and foot-off) were identified from the heel, toe, and sacrum marker kinematic traces as described by Zeni et al.^21^. Gait trials were then partitioned into individual gait cycles. Due to the asymmetrical motor impairments in our CP group, gait cycles of the most affected side were retained for analyses, while the right side was kept for the TD group^22^. The most affected side was determined based on the results of the spasticity, passive range of motion assessments, and kinematics curves. The first 10 gait cycles of each participant/condition (i.e., each surface) were used for the rest of the analysis. For the participants for whom we did not obtain sufficient gait cycles (< 10 gait cycles for each gait condition; CP: n = 6, TD: n = 12), all available gait cycles were used (mean ± standard deviation: 8 ± 1 gait cycles/gait condition).

### Kinematics parameters

Hip, knee, and ankle sagittal joint kinematics, and walking speed were calculated. The joint’s minimal, maximal, and range of motion angles during the stance and swing phase were determined. Gait speed was normalized to the participant’s leg length^23^. The values of the participant’s most representative gait cycle for each condition were used for statistical analysis. The most representative gait cycle was selected according to the gait cycle with the minimal root mean square difference with the mean for the hip, knee, and ankle sagittal plane kinematics^24,25^.

### Inter-joint coordination calculation

The inter-joint coordination was evaluated with the continuous relative phase (CRP) method, which is the calculation of the phase angle difference between the two joints. The hip, knee, and ankle phase angles in the sagittal plane were calculated according to Lamb and Stöckl’s approach^26^, as described elsewhere^13^. A padding technique, i.e., conserving data frames before and after the gait cycle of interest, was used to minimize data distortion caused by the Hilbert Transform^27^. The CRP curves indicate the in-phase/out-of-phase coupling relationships between the two joints (i.e., inter-joint coordination), for which a value of 0° indicates joints that are moving fully in phase with each other, whereas a value of 180° refers to a fully out-of-phase coupling^28^. A CRP curve of the knee-hip and ankle-knee joint pairs was calculated for each participant’s gait cycle. The mean absolute relative phase (MARP), which is the mean of the ensemble CRP curves, was calculated for each participant/condition. Inter-joint coordination values closer to zero indicate a more in-phase strategy, which has been interpreted previously as a more rigid gait pattern^13,29^.

### Statistical analyses

Age, body mass, and height were compared between groups with unpaired t-tests or Wilcoxon signed-rank tests for parametrically or non-parametrically distributed data (i.e., normality and homogeneity of variance), respectively. To test our hypothesis, each dependant variable (joint kinematics, gait speed, and inter-joint coordination metrics) were tested for between subject variable group (CP, TD) and within-subject variable surface condition (even, medium, high) main effect, and group × condition interaction using a 2-way mixed analysis of variance (ANOVA). For the continuous variables (i.e., joint kinematics and inter-joint coordination metrics), ANOVAs were performed using the Statistical Parametric Mapping (SPM) toolbox (spm1d, v.M.0.4.10)^30^. In the case of a significant group × condition interaction or condition main effect, post-hoc comparisons with Bonferroni correction were computed (i.e., n = 3 for surface comparison). The clusters’ (i.e., multiple adjacent points of the SPM{t} curve that exceed the critical threshold) *p*-values were reported^31^. Cohen’s d effect size was calculated for each cluster’s point, and the average effect size was reported as the cluster’s effect size. Only clusters lasting 5% of the gait cycle or more were discussed^32^. All statistical analysis steps were run in custom-made Matlab scripts (v2022b, The Mathworks, Inc. Natick, USA).

## Results

A total of 17 children with spastic CP (Gross Motor Function Classification System (GMFCS) I: n = 13, GMFCS II: n = 4; Unilateral CP: n = 7, Bilateral CP: n = 10) and 17 TD children were recruited. Anthropometrics and characteristics of the included participants are reported in Table 1. There were no differences between the two groups in terms of age (*p* = 0.436), body mass (*p* = 0.863), and height (*p* = 0.587). Among the 15 participants with CP for whom range of motion was measured, 8 of them exhibited plantar flexor contractures (only knee flexed: n = 1, only knee extended: n = 3, knee extended and flexed: n = 4) (see Table 2). Joint contracture details for each participant are presented in Supplementary Table S1. Concerning normalized gait speed, no group × condition interaction (*p* = 0.887) or condition effect (*p* = 0.130) was observed (CP: even: 0.41 ± 0.10; medium: 0.36 ± 0.11; high: 0.34 ± 0.14; TD: even: 0.50 ± 0.07; medium: 0.47 ± 0.05; high: 0.44 ± 0.09). All kinematics and inter-joint coordination parameters showed at least one significant moment during the gait cycle with a group × condition interaction or condition main effect. The detailed results of the ANOVAs are presented in the Supplementary Figure S1. Each joint’s minimal, maximal, and range of motion angles during the stance and swing phase are also reported in the Supplementary Table S2.

**Table 1:** Anthropometrics and characteristics of participants.

Group	*n*	CP type	Age (years)	Weight (kg)	Height (cm)	Sex	Orthosis worn	Past medical interventions
CP	17	U: 7 B: 10	11.48 (3.49)	36.39 [29.93, 42.84]	143.26 (17.13)	M: 11 F: 6	sAFO: 5 FO: 1None: 11	BTX: 2 S: 1
TD	17	N/A	10.39 (4.50)	40.16 [29.24, 51.07]	139.41 (23.30)	M: 8 F: 9	N/A	N/A
*p*	–	–	0.436	0.863	0.587	–	–	

Mean (standard deviation) or median [95% confidence interval] for parametrically and non-parametrically distributed data (i.e., normality and homogeneity of variance), respectively. Anthropometrics presented for children with cerebral palsy (CP) and their typically developing (TD) peers. *p*-Values (*p*) for age, weight and height differences between groups are presented. Sample size (n), CP type; unilateral (U) or bilateral (B), or not applicable (N/A), sex; female (F) and male (M), the orthosis worn; foot orthotic (FO) or solid ankle–foot orthosis (sAFO), and the past medical interventions within the last 12 months; botulinum toxin injection (BTX) and surgery (S) are reported.

**Table 2:** Joint contractures according to the goniometer measurements.

	Hip extension	Knee extension	Ankle dorsiflexion (knee flexed)	Ankle dorsiflexion (knee extended)
Mean (SD)	25.87 (3.14)	− 0.53 (1.30)	11.67 (7.48)	7.13 (7.11)
*n*	0	0	5	7

Mean (standard deviation) passive range of motion is presented for the 15/17 participants with cerebral palsy assessed. Values were compared to the age-normative passive range of motion reported by Mudge et al.^50^ (knee extension and ankle dorsiflexion) and Sankar et al.^51^ (hip extension). A contracture was reported if the range of motion was 6°, 4°, and 8° lower than the minimum normative value for the hip extension^51^, knee extension^52^ and ankle dorsiflexion^53^, respectively. A negative value represents flexion angle for the hip and knee extension, and plantarflexion angle for the ankle dorsiflexion. The distribution (*n*) of joint contracture is presented for each measurement.

### Kinematics parameters

#### Hip joint

**Figure 2:**
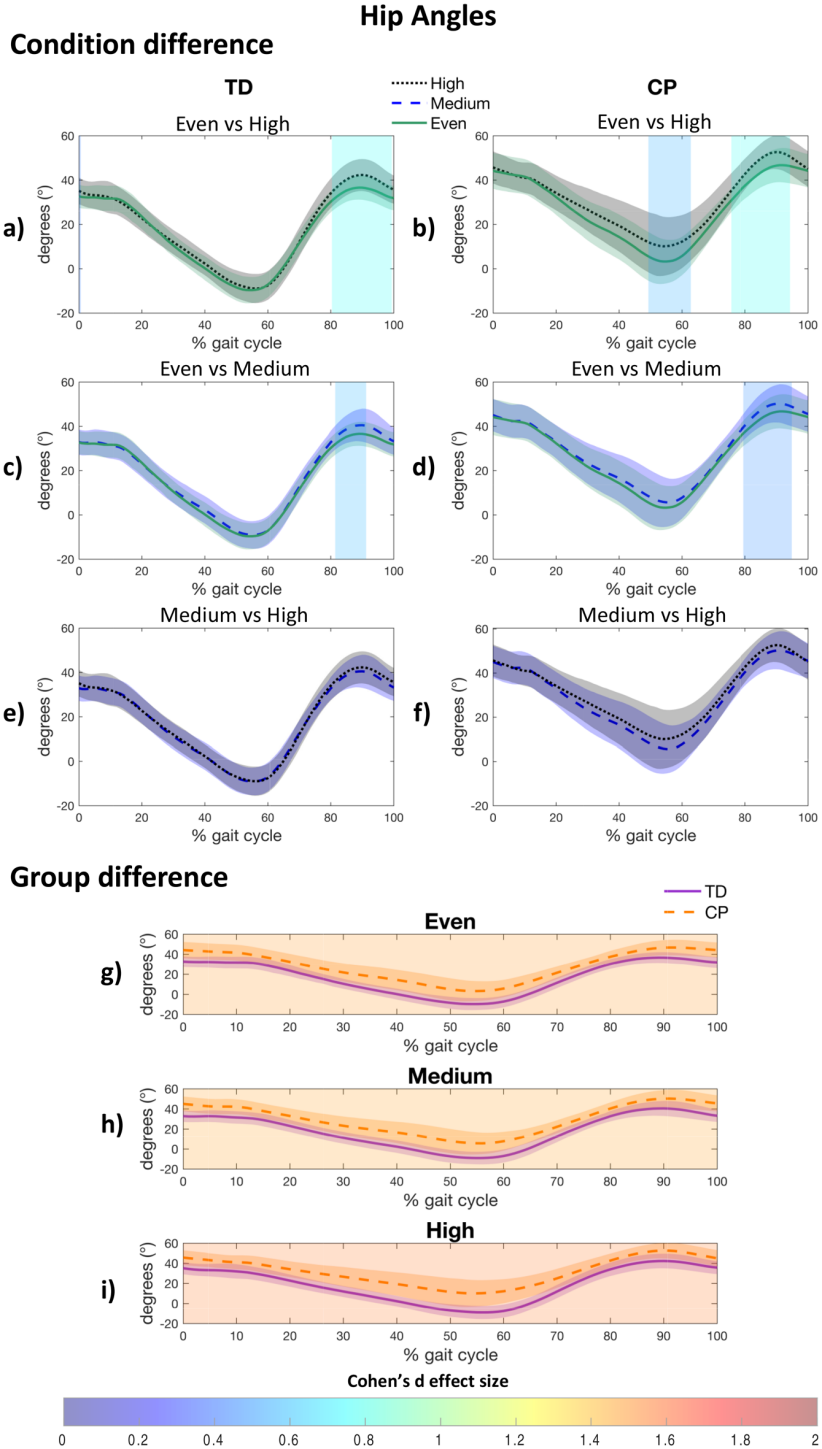
Post-hoc results for within group (**a**–**f**) and between group (**g**–**i**) difference for the hip kinematics. A patch represents a significant difference between curves and is colored by the associated Cohen’s d effect size. CP, Cerebral palsy; TD, Typically developing.

In comparison with the even surface, both groups increased their hip flexion in late swing when walking on the medium (CP: 80–95%, d = 0.467, *p* < 0.001; TD: 81–91%, d = 0.559, *p* = 0.001) and high (CP: 76–94%, d = 0.776, *p* < 0.001; TD: 80–99%, d = 0.759, *p* < 0.001) uneven surface (Fig. 2a–d). No difference in hip flexion was observed between medium and high uneven surface (Fig. 2e,f). The CP group also revealed decreased hip extension during late stance on high level (49–63%, d = 0.549, *p* < 0.001) compared to even surface (Fig. 2b). This adaptation was not present in the TD group (Fig. 2a). Regardless of the level of unevenness, children with CP showed less hip extension than the TD group during the entire gait cycle (d > 1.455, *p* < 0.001) (Fig. 2g–i), marked by greater maximal flexion during initial contact (CP: even: 44.6° ± 8.2°; medium: 45.2° ± 7.4°; high: 45.8° ± 7.4°; TD : even: 33.8° ± 5.3°; medium: 33.7° ± 5.7°; high: 35.4° ± 6.4°), reduced maximal extension during late stance (CP: even: 2.4° ± 9.5°; medium: 4.6° ± 10.3°; high: 8.5° ± 13.3°; TD: even: − 10.1° ± 6.1°; medium: − 9.3° ± 6.4°; high: − 9.4° ± 6.7°), and increased maximal flexion during the swing phase (CP: even: 47.3° ± 8.1°; medium: 51.0° ± 8.8°; high: 52.9° ± 7.9°; TD: even: 37.0° ± 5.6°; medium: 40.8° ± 7.5°; high: 42.9° ± 7.6°) (see Supplementary Table S2 online**)**.

#### Knee joint

**Figure 3:**
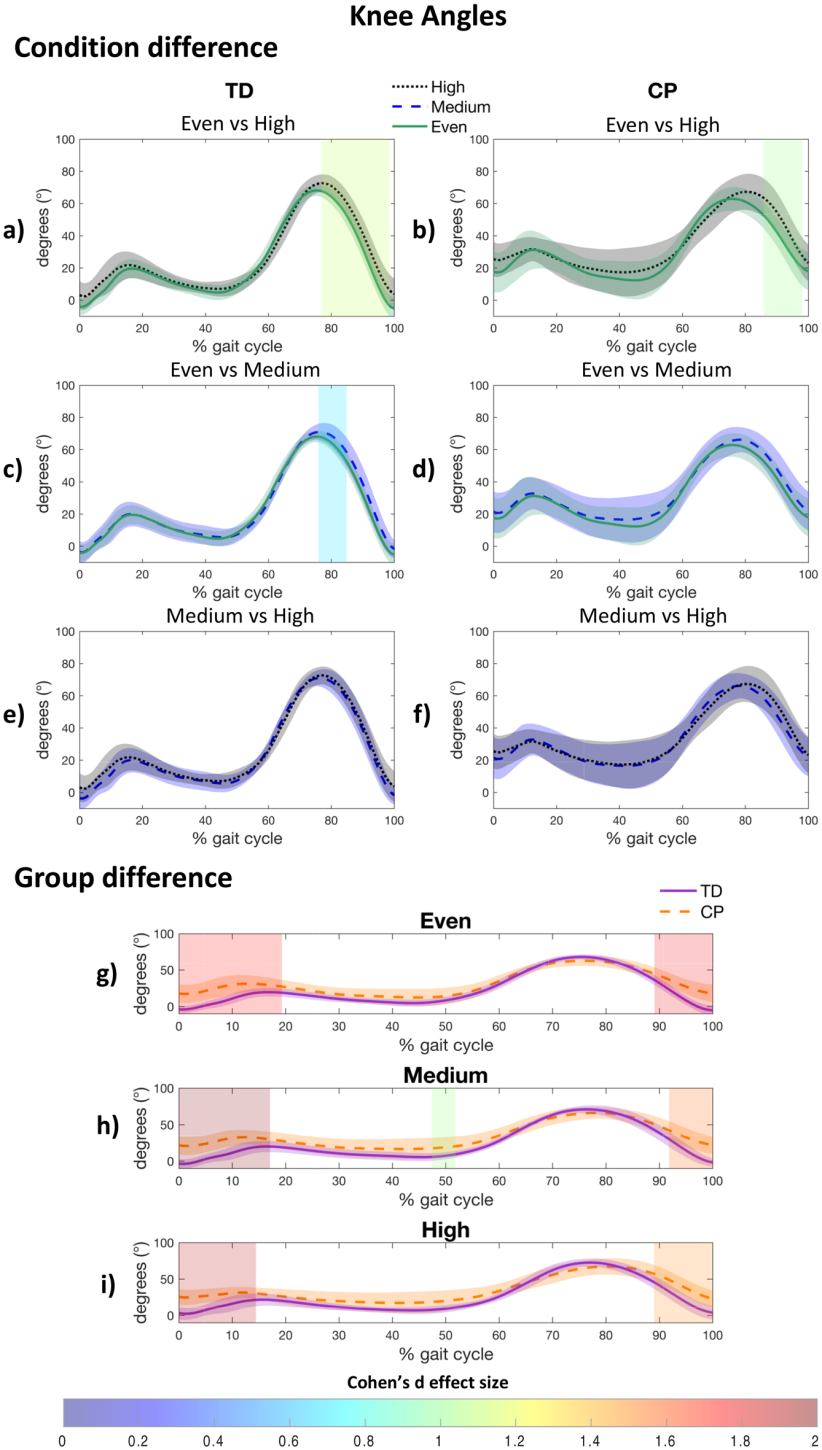
Post-hoc results for within group (**a**–**f**) and between group (**g**–**i**) difference for the knee kinematics. A patch represents a significant difference between curves and is colored by the associated Cohen’s d effect size. CP, cerebral palsy; TD, typically developing.

Both groups decreased their knee extension in late swing when walking on the high uneven surface, in comparison with the even surface (CP: 86–98%, d = 1.018, *p* < 0.001; TD: 77–98%, d = 1.112, *p* < 0.001) (see Fig. 3a,b), while only the TD group decreased their knee extension during late swing on the medium uneven surface (TD: 76–85%, d = 0.680, *p* = 0.001) (Fig. 3c,d). No differences in knee kinematics were observed between medium and high levels (Fig. 3e,f). Compared with the TD group, less knee extension in the CP group was observed regardless of the level of unevenness during loading response (even: 0–19%, d = 1.725, *p* = 0.001; medium: 0–17%, d = 1.982, *p* = 0.004; high: 0–14%, d = 1.893, *p* = 0.005) and late swing (even: 89–100%, d = 1.773, *p* = 0.012; medium: 92–100%, d = 1.560, *p* = 0.028; high: 89–100%, d = 1.484, *p* = 0.013) (Fig. 3g–i), as marked by the greater knee flexion prior the foot strike (CP: even: 17.9° ± 12.1°; medium: 21.9° ± 12.4°; high: 23.1° ± 11.7°; TD: even: − 5.4° ± 7.0°; medium: − 1.9° ± 5.8°; high: 3.8° ± 9.9°) (see Supplementary Table S2 online).

#### Ankle joint

**Figure 4:**
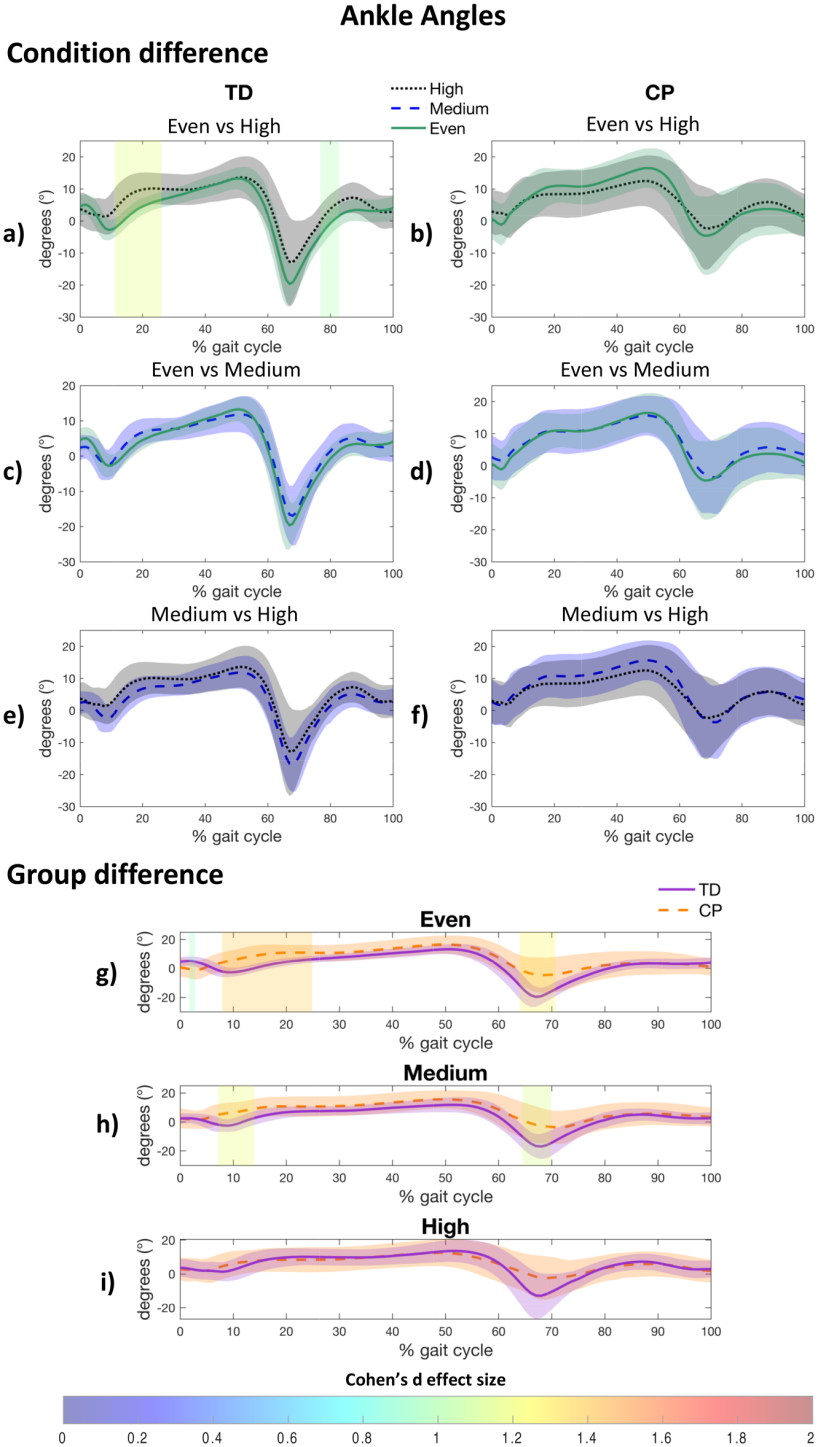
Post-hoc results for within group (**a**–**f**) and between group (**g**–**i**) difference for the ankle kinematics. A patch represents a significant difference between curves and is colored by the associated Cohen’s d effect size. CP, Cerebral palsy; TD, Typically developing.

At the ankle joint, only the TD group increased dorsiflexion during stance (11–26%, d = 1.167, *p* < 0.001) and swing (77–83%, d = 0.989, *p* = 0.003) when walking on the high uneven surface, in comparison with the even surface (Fig. 4a). This increase was not observed when comparing the even surface to the medium uneven surface (Fig. 4c) or the medium uneven surface to the high uneven surface (Fig. 4e). On the even surface and the medium uneven surface, children with CP showed greater ankle dorsiflexion than the TD group during stance (even: 8–25%, d = 1.394, *p* = 0.001; medium: 7–14%, d = 1.189, *p* = 0.029) and swing phase (even: 64–71%, d = 1.273, *p* = 0.028; medium: 64–70%, d = 1.122, *p* = 0.035) (Fig. 4g,h), thereby demonstrating a smaller range of motion during stance (CP: even: 24.0° ± 5.1°; medium: 20.8° ± 5.2°; TD: even: 30.1° ± 3.9°; medium: 26.9° ± 7.1°) and swing phase (CP: even: 13.6° ± 7.7°; medium: 14.2° ± 9.3°; TD: even: 26.7° ± 6.9°; medium: 24.7° ± 7.1°) (see Supplementary Table S2 online).

### Inter-joint coordination

#### Knee-hip coordination

**Figure 5:**
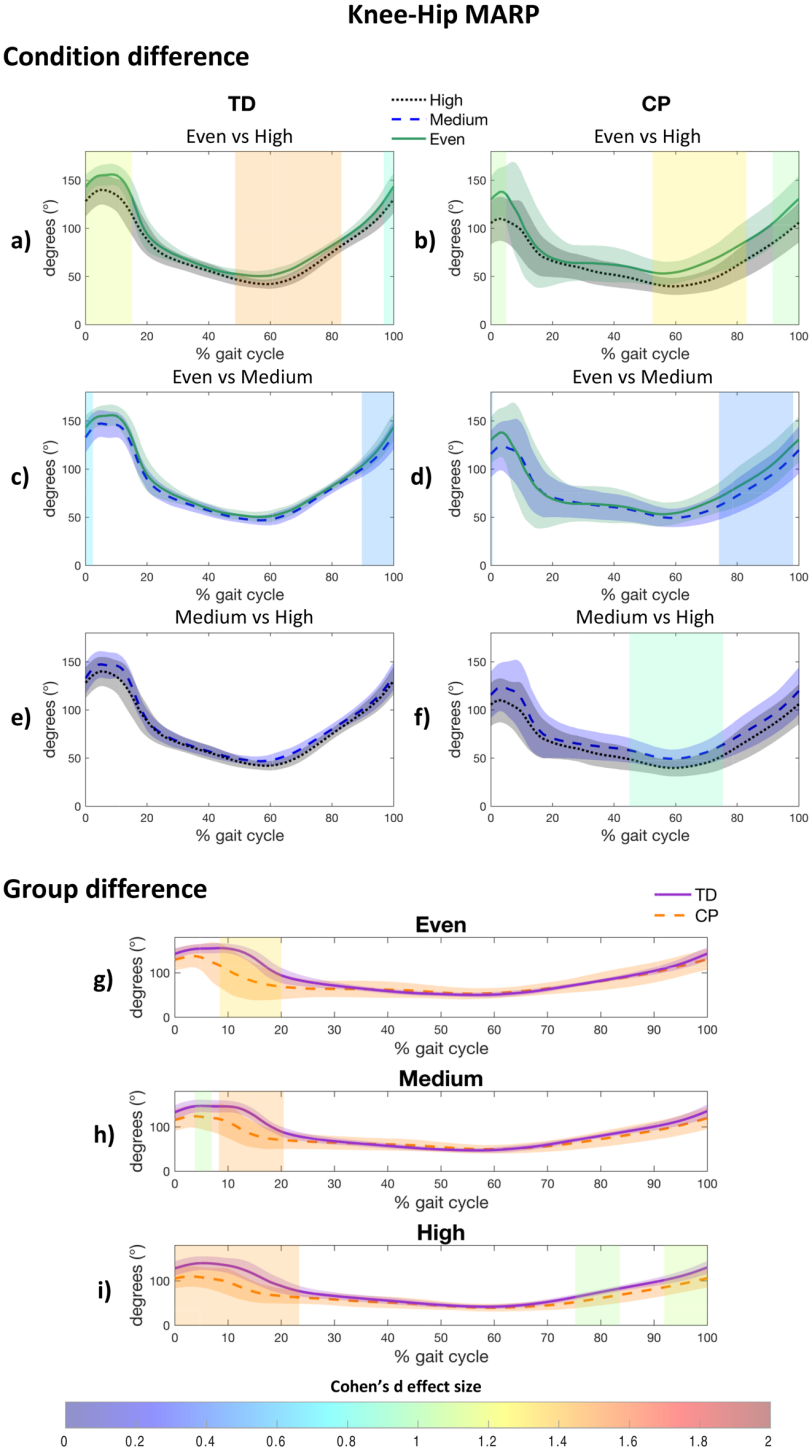
Post-hoc results for within group (**a**–**f**) and between group (**g**–**i**) difference for Knee-Hip Mean Absolute Relative Phase (MARP). A patch represents a significant difference between curves and is colored by the associated Cohen’s d effect size. CP, Cerebral palsy; TD, Typically developing.

Both groups used a greater knee-hip in-phase coordination pattern during loading response (CP: 0–5%, d = 1.036, *p* = 0.012; TD: 0–15%, d = 1.161, *p* < 0.001), and terminal stance/swing phase (CP: 53–83%, d = 1.284, *p* < 0.001 and 92–100%, d = 0.992, *p* = 0.006; TD: 49–83%, d = 1.460, *p* < 0.001), when walking on the high uneven surface, in comparison with the even surface (Fig. 5a,b). This coordination pattern was also observed in both groups on the medium uneven surface during the end of swing phase (CP: 74–98%, d = 0.458, *p* < 0.001; TD: 90–100%, d = 0.492, *p* < 0.001), in comparison with the even surface (Fig. 5c,d). When comparing medium and the high uneven surface, only the CP group showed a greater knee-hip in-phase coordination pattern when the unevenness is increased (45–75%, d = 0.895, *p* < 0.001) (Fig. 5e,f). The CP group had a more in-phase coordination pattern during early stance regardless of the unevenness level (even: 8–20%, d = 1.320, *p* = 0.015; medium: 4–7%, d = 1.034, *p* = 0.046 and 8–20%, d = 1.470 *p* = 0.014; high: 0–23%, d = 1.456, *p* = 0.001), and during swing phase on the high uneven surface (75–84%, d = 1.035, *p* = 0.029 and 92–100%, d = 1.091, *p* = 0.030) (Fig. 5g–i).

#### Ankle-knee coordination

**Figure 6:**
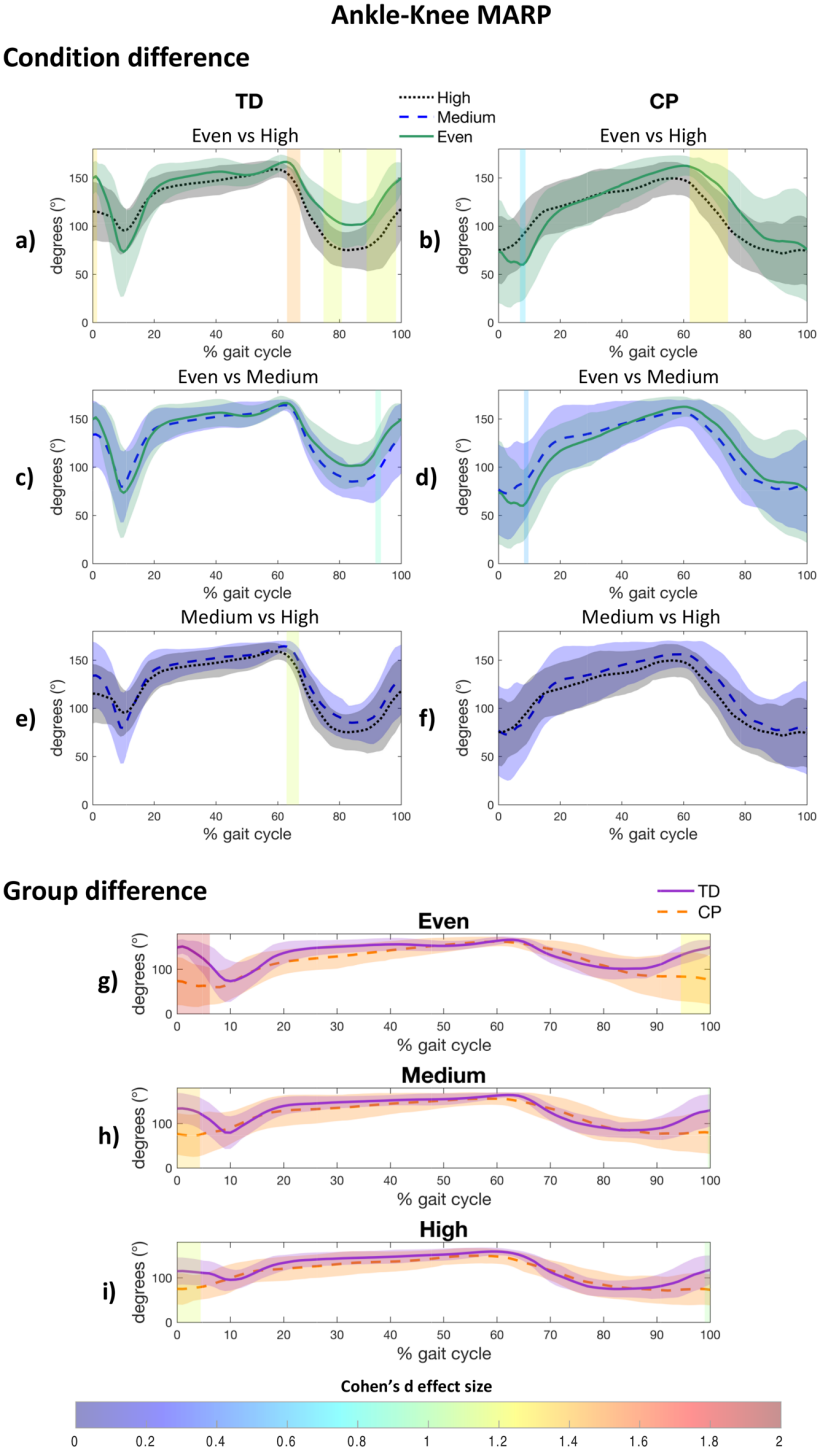
Post-hoc results for within group (**a**–**f**) and between group (**g**–**i**) difference for Ankle-Knee Mean Absolute Relative Phase (MARP). A patch represents a significant difference between curves and is colored by the associated Cohen’s d effect size. CP, Cerebral palsy; TD, Typically developing.

Regarding the ankle-knee coordination, greater in-phase coordination was observed in both groups during swing when walking on the high uneven surface, compared to the even surface (CP: 62–74%, d = 1.255, *p* < 0.001; TD: 75–81%, d = 1.173, *p* = 0.003, and 89–98%, d = 1.179, *p* < 0.001) (Fig. 6a,b). This greater in-phase coordination was not observed in both groups on the medium and high uneven surface, in comparison with the even surface (Fig. 6c–f). Compared with the TD group, CP had a more in-phase coordination pattern in early stance and late swing when walking on the even surface (early stance: 0–6%, d = 1.848, *p* = 0.019; late swing: 94–100%, d = 1.239, *p* = 0.023), and early stance when walking on the medium uneven surface (early stance: 0–5%, d = 1.352, *p* = 0.034) and high uneven surface (early stance: 0–5%, d = 1.157, *p* = 0.034) (Fig. 6g–i).

## Discussion

This study investigated sagittal lower-limb kinematics and inter-joint coordination patterns (MARP) in children with CP and their TD peers during gait on even and two levels of uneven surface (medium and high), and assessed differences between groups. Three findings warrant highlighting. First, uneven surfaces induced mostly proximal (i.e., hip and knee) gait adaptations rather than distal (i.e., ankle). Second, children with CP exhibited different adaptations compared to their TD peers, notably a decreased hip extension during late stance, and the absence of an increase in ankle dorsiflexion during mid-stance. Third, children with CP used a more in-phase knee-hip coordination strategy than their TD peers during swing phase, only when walking on the high uneven surface.

Consistent with our hypothesis, uneven surfaces induced joint flexion adaptations in children with CP, more precisely greater hip and knee flexion during late swing (Figs. 2b and 3b, respectively). These adaptations have been reported by previous work as a shortening-leg strategy that facilitates minimal toe clearance, which is required to prevent trips and falls^33,34^. Uneven surface induces a reduced hip extension (approximately 6°) during late stance in the CP group (only on high uneven surface), while this adaptation is not apparent in the TD group (Fig. 2a,b). This reduced hip extension is likely not related to hip flexion contracture, as no hip flexion contracture was reported among all the included participants (Table 2). Moreover, this adaptation does not appear to be associated with a difference in gait speed^35^, as our analysis of variance indicates that children with CP and those with TD adjust their gait speed in a similar manner across surfaces. This CP-specific adaptation, implemented at a moment in the gait cycle when the center of mass reaches its maximal height, may be a strategy aimed at lowering the overall position of the body's center of mass closer to the ground to increase stability and reduce the likelihood of losing balance^36^. Moreover, a lack of knee extension at loading response and late swing has been observed in the CP compared to the TD group, regardless of the surface. This gait deviation has been previously related to the presence of knee flexion contractures^37^. However, passive range of motion assessment revealed no knee flexion contracture for all participants with CP (Table 2), supporting an interpretation where gait adaptations are implemented to maintain joint stability during weight shifting (i.e., late swing and loading response), as a compensation for the lack of neural control^38^.

Distally, a high level of unevenness induced greater ankle dorsiflexion during mid-stance in comparison with even surface in the TD group only (Fig. 4a,b,i). The inability of children with CP to appropriately adjust ankle movements to comply with uneven surfaces has been also reported by other studies^8,33^, and has been attributed to motor control impairments^8^, gastrocnemius contractures, and spasticity^33^. In this study, the lack of ankle dorsiflexion adaptation (Fig. 4b,d,f) may be related to the observation that over half of the participants with CP exhibited plantar flexor contracture, and 5 participants utilized solid ankle–foot orthoses during walking. Moreover, less plantar flexion during push-off is observed in the CP group on even and medium uneven surfaces, compared to the TD group (Fig. 4g,h). The relationship between plantar flexor weakness and reduced pushing power in people with central motor injuries such as children with CP is known^39^. Weakened thrust strength may result in a lack of stability during rolling^40,41^ and a limited vertical and forward component of propulsion^42–46^.

In line with our hypothesis, the CP group implemented a more in-phase inter-joint coordination strategy when walking on uneven surfaces, specifically at initial contact and swing phase for the knee-hip joint pair (Fig. 5b). These results are in accordance with the knee-hip in-phase pattern that has been reported during these gait phases in elderly adults when walking on uneven brick walkways^29^. This joint-coordinated behavior when walking on more challenging surfaces has been related to a ‘cautious’ and rigid strategy. Also, children with CP presented a more in-phase knee-hip coordination pattern than their TD peers during early stance phase (Fig. 5g–i), due to a lack of knee flexion increase during the loading response. Indeed, compared to their TD peers, children with CP had a more flexed knee at initial contact, but exhibited less flexion increase during the loading phase (Fig. 3b). This lack of knee flexion during loading phase has been related to several reasons, notably quadriceps weakness^37^ or spasticity^3^. Moreover, the most challenging (i.e., high uneven surface) surface highlights impaired knee-hip coordination during late swing in children with CP (Fig. 5b,i). Indeed, children with CP are not able to isolate knee motion independently from the hip in an out-of-phase manner (i.e., extending the knee while flexing the hip) as their TD peers, which has been related to an impaired selective motor control^13,47,48^. This result supports that a surface with high level of unevenness may detect motor impairments that are not apparent on even surfaces, which points out the potential benefits of integrating uneven surfaces in clinical gait analysis for a more exhaustive and ecological assessment of gait.

For the ankle-knee joint pair, our findings showed a more in-phase coordination strategy in children with CP during early stance, regardless of the surface (Fig. 6g–i). Indeed, at initial contact, TD children tend towards a more in-phase coordination (i.e., knee flexion during the first rocker), followed by a quick switch towards an out-of-phase coordination (i.e., knee flexion during the second rocker). This coordination oscillation during the loading response was almost inexistent in children with CP (Fig. 6b,d,f) and is primarily due to the absence of the first rocker and the lacking knee flexion increase (Fig. 3g–i). The same absence of coordination oscillation was present during late swing (i.e., ~ 90% of the gait cycle), when the TD group increased their ankle-knee coordination in an out-of-phase manner (due to the quick knee extension increase), and the CP group remains stable (due to the lack of knee extension increase).

This research has clinical implications for both rehabilitation and assessment. Our findings show potential benefits of integrating uneven surfaces into clinical gait analysis to detect strategies used during walking in typical daily environments, revealing gait limitations that are not elicited by conventional analysis on a flat surface. Moreover, identifying gait deviations in children with CP on uneven surfaces provides valuable information for developing targeted rehabilitation strategies. By training and assessing gait on uneven surfaces, children with CP can develop better skills to navigate real-world environments. This can lead to improved overall mobility, greater independence, and enhanced quality of life. Finally, this study provides reference values for laboratories wishing to undertake similar assessments.

Our conclusions must be interpreted with awareness of a few limitations. First, our study is limited to the analysis of movement in the sagittal plane, which may potentially overlook gait deviations that occur in other planes (e.g., hip abduction and rotation). Second, each participant walked at their self-selected speed to allow ecological assessment (i.e., gait speed they would normally opt for during daily locomotion), and gait speed may influence kinematics^35^ and inter-joint coordination patterns^49^. However, analysis of gait speed showed no group × condition interaction, suggesting that the reported kinematics and inter-joint coordination adaptations to uneven surfaces are not attributable to differences in gait speed. Third, we only included children with CP with a GMFCS level I and II, and more than 75% were classified as GMFCS level I. This predominance limits the generalizability of our results to children with GMFCS level II. Also, 2 participants received botulinum toxin injections and 1 participant underwent an orthopedic surgery (femoral plate implant) 5 months and 4.5 months before the gait analysis, respectively. Fourth, further studies, including electromyography and kinetic data are needed to better interpret the different strategies used by children with CP when walking on uneven surfaces; however, the latter may be particularly complex to measure via force plates beneath an uneven surface (variable, unknown distance between point of contact on surface and top of the force plate). Finally, future research with larger sample size is warranted to explore potential differences in adaptations between unilateral and bilateral CP, offering valuable insights for the development of nuanced rehabilitation strategies tailored to their distinct needs.

This study allows for a better understanding of how children with spastic CP adapt their gait on uneven surfaces. Moreover, gait analysis on uneven surfaces enables the assessment of motor impairments that are not present on even surface (e.g., impaired knee-hip coordination during swing), promoting the interest in using more ecological approaches for gait assessment. Future studies should explore if rehabilitation therapy on uneven surfaces can contribute to enhancing walking abilities in children with CP on challenging surfaces.

### Supplementary Information

Supplementary Information.

## Data Availability

Code and data supporting the results reported in the article can be provided by the corresponding author.
