# Supplementary material for: The effect of uneven surfaces on inter-joint coordination during walking in children with cerebral palsy
Source: Sci Rep. 2023 Dec 8;13:21779. doi: 10.1038/s41598-023-49196-w (PMC10709314; doi:10.1038/s41598-023-49196-w)
Supplement: Supplementary file 1 — Supplementary Information. [file 41598_2023_49196_MOESM1_ESM.docx]

**Supplementary Information**

**The effect of uneven surfaces on inter-joint coordination during walking in children with cerebral palsy**

Dussault-Picard C ^a,b^, Cherni Y ^a,b^, Ferron A ^b,c^, Robert M.T, Dixon P.C ^a,b,e^

**Supplementary Figure S1.** Group effect (column 1), condition effect (column 2), and group × condition interaction (column 3) are presented for each variable. Significant part of the gait cycle is presented in gray. MARP, mean absolute relative phase.

**Supplementary Table S1.** Passive range of motion and normative values

| **Participant** | **Norm**  **(°)** | **Hip extension**  **(°)** | **Norm**  **(°)** | **Knee extension**  **(°)** | **Norm**  **(°)** | **Ankle dorsiflexion (knee flexed)**  **(°)** | **Norm**  **(°)** | **Ankle dorsiflexion (knee extended)**  **(°)** |
| --- | --- | --- | --- | --- | --- | --- | --- | --- |
| **CP1** | 20.3-22.8 | 22 | -3-5 | -1 | 17.4-30 | -8 * | 13.9-23.7 | -5 * |
| **CP2** | 17.5-19.71 | 27 | 1-9 | 0 | 23-35 | 10 * | 16.8-29 | 5 * |
| **CP3** | 17.5-19.71 | 26 | -2-10 | 0 | 20.5-34.3 | 16 | 17.5-26.3 | 15 |
| **CP4** | 13.9-16.5 | 28 | -3-5 | -3 | 17.4-30 | 5 * | 13.9-23.7 | -4 * |
| **CP5** | 20.3-22.8 | 24 | -3-5 | 0 | 17.4-30 | 11 | 13.9-23.7 | 7 |
| **CP6** | 20.3-22.8 | 29 | -3-5 | 0 | 17.4-30 | 19 | 13.9-23.7 | 15 |
| **CP7** | 17.5-19.71 | 26 | 1-9 | -2 | 23-35 | 13 * | 16.8-29 | 11 |
| **CP8** | 19.7-23.0 | 24 | -2-10 | 0 | 20.5-34.3 | 5 * | 17.5-26.3 | 5 * |
| **CP9** | 19.7-23.0 | 26 | -2-10 | -3 | 20.5-34.3 | 15 | 17.5-26.3 | 13 |
| **CP10** | 13.9-16.5 | 25 | -3-5 | 0 | 17.4-30 | 17 | 13.9-23.7 | 8 |
| **CP11** | 13.9-16.5 | 21 | -3-5 | 0 | 17.4-30 | 14 | 13.9-23.7 | 6 |
| **CP12** | 13.9-16.5 | 18 | -3-5 | 2 | 17.4-30 | 10 | 13.9-23.7 | -1 * |
| **CP13** | 19.1-22.5 | 24 | 1-9 | 0 | 23-35 | 25 | 16.8-29 | 20 |
| **CP14** | 19.7-23.0 | 30 | -2-10 | 0 | 20.5-34.3 | 13 | 17.5-26.3 | 9 * |
| **CP15** | 17.5-19.71 | n/a | -2-10 | n/a | 20.5-34.3 | n/a | 17.5-26.3 | n/a |
| **CP16** | 13.9-16.5 | 23 | -3-5 | -1 | 17.4-30 | 10 | 13.9-23.7 | 3 * |
| **CP17** | 13.9-16.5 | n/a | -3-5 | n/a | 17.4-30 | n/a | 13.9-23.7 | n/a |
| **Mean (SD)** | n/a | 25.87 (3.14) | n/a | -0.53 (1.30) | n/a | 11.67 (7.48) | n/a | 7.13 (7.11) |

Mean (standard deviation) are reported for each measurement (degrees). Range of normative values is also presented according to the age (knee extension ^52^ and ankle dorsiflexion ^53^) or age and sex (hip extension ^51^) of each participant. A negative value represents flexion angle for the hip and knee extension, and plantarflexion angle for the ankle dorsiflexion. Asterisk (*) represents a contracture, according to the normative data. Norm, normative data; SD, standard deviation, n/a, not available.

**Supplementary Table S2.** Minimal, maximal and range angles of each joint during stance and swing phases.

|  |  |  | **Stance** | | **Swing** | |
| --- | --- | --- | --- | --- | --- | --- |
|  |  |  | **TD** | **CP** | **TD** | **CP** |
| **Even** | **Hip** | **min** | -10.05 ± 6.14 | 2.42 ± 9.54 | 0.54 ± 5.80 | 11.12 ± 8.67 |
|  |  | **max** | 33.67 ± 5.34 | 44.63 ± 8.17 | 36.96 ± 5.63 | 47.28 ± 8.06 |
|  |  | **rom** | 43.72 ± 6.31 | 42.21 ± 6.59 | 36.42 ± 5.57 | 36.16 ± 6.64 |
|  | **Knee** | **min** | -4.69 ± 4.98 | 9.64 ± 10.79 | -5.38 ± 7.04 | 17.93 ± 12.13 |
|  |  | **max** | 48.13 ± 4.73 | 47.97 ± 7.99 | 68.69 ± 3.93 | 64.01 ± 7.63 |
|  |  | **rom** | 52.82 ± 5.23 | 38.33 ± 10.11 | 74.07 ± 8.66 | 46.08 ± 15.83 |
|  | **Ankle** | **min** | -15.75 ± 6.49 | -6.09 ± 6.36 | -21.48 ± 5.80 | -8.72 ± 12.26 |
|  |  | **max** | 14.38 ± 3.11 | 17.94 ± 5.95 | 5.21 ± 3.95 | 4.92 ± 7.45 |
|  |  | **rom** | 30.14 ± 3.93 | 24.03 ± 5.12 | 26.69 ± 6.93 | 13.64 ± 7.69 |
| **Medium** | **Hip** | **min** | -9.33 ± 6.40 | 4.59 ± 10.31 | 1.17 ± 6.55 | 13.38 ± 8.95 |
|  |  | **max** | 33.68 ± 5.72 | 45.20 ± 7.37 | 40.81 ± 7.53 | 51.01 ± 8.82 |
|  |  | **rom** | 43.01 ± 4.72 | 40.61 ± 7.76 | 39.64 ± 7.64 | 37.64 ± 7.85 |
|  | **Knee** | **min** | -4.33 ± 6.55 | 13.99 ± 13.88 | -1.91 ± 5.83 | 21.90 ± 12.39 |
|  |  | **max** | 47.26 ± 3.94 | 49.21 ± 11.00 | 71.63 ± 5.99 | 68.35 ± 7.79 |
|  |  | **rom** | 51.59 ± 7.83 | 35.22 ± 11.03 | 73.53 ± 6.14 | 46.45 ± 13.09 |
|  | **Ankle** | **min** | -12.84 ± 8.60 | -3.51 ± 6.63 | -18.72 ± 8.38 | -7.47 ± 12.04 |
|  |  | **max** | 14.03 ± 2.88 | 17.24 ± 6.91 | 5.99 ± 4.41 | 6.73 ± 7.66 |
|  |  | **rom** | 26.88 ± 7.09 | 20.75 ± 5.19 | 24.72 ± 7.14 | 14.20 ± 9.33 |
| **High** | **Hip** | **min** | -9.42 ± 6.69 | 8.49 ± 13.30 | 1.23 ± 6.99 | 16.68 ± 12.46 |
|  |  | **max** | 35.41 ± 6.40 | 45.84 ± 7.41 | 42.88 ± 7.55 | 52.89 ± 7.91 |
|  |  | **rom** | 44.83 ± 5.87 | 37.34 ± 10.36 | 41.65 ± 8.35 | 36.22 ± 11.68 |
|  | **Knee** | **min** | 0.02 ± 5.61 | 15.01 ± 13.97 | 3.82 ± 9.88 | 23.13 ± 11.72 |
|  |  | **max** | 46.89 ± 4.54 | 46.56 ± 9.51 | 73.30 ± 5.56 | 69.54 ± 10.66 |
|  |  | **rom** | 46.87 ± 7.06 | 31.54 ± 9.81 | 69.48 ± 10.74 | 46.41 ± 13.58 |
|  | **Ankle** | **min** | -11.26 ± 8.77 | -3.81 ± 7.66 | -15.75 ± 9.81 | -6.31 ± 11.00 |
|  |  | **max** | 16.42 ± 6.38 | 14.16 ± 7.77 | 7.90 ± 4.98 | 6.27 ± 7.42 |
|  |  | **rom** | 27.68 ± 5.47 | 17.97 ± 5.92 | 23.65 ± 9.32 | 12.58 ± 6.20 |

**Abbreviations.** cerebral palsy (CP), minimal (min), maximal (max), range of motion (rom), typically developing (TD).
